# Development of Primer Panels for Amplicon Sequencing of Human Parainfluenza Viruses Type 1 and 2

**DOI:** 10.3390/ijms252313119

**Published:** 2024-12-06

**Authors:** Oula Mansour, Artem V. Fadeev, Alexander A. Perederiy, Daria M. Danilenko, Dmitry A. Lioznov, Andrey B. Komissarov

**Affiliations:** 1 Smorodintsev Research Institute of Influenza, 197376 Saint Petersburg, Russia; olamansor1995@gmail.com (O.M.); afadeew@gmail.com (A.V.F.); gilagalex@gmail.com (A.A.P.); daria.baibus@gmail.com (D.M.D.); dlioznov@yandex.ru (D.A.L.); 2 Department of Infectious Diseases and Epidemiology, Pavlov First Saint Petersburg State Medical University, 197022 Saint Petersburg, Russia

**Keywords:** human parainfluenza virus, hPIV1, hPIV2, primer panels, whole-genome amplification, phylogenetic tree

## Abstract

Human parainfluenza viruses (hPIVs) are major contributors to respiratory tract infections in young children worldwide. Despite their global significance, genomic surveillance of hPIV1 and hPIV2 had not previously been conducted in Russia. This study aimed to develop a robust amplicon-based sequencing protocol for these viruses. The designed primer sets were tested on clinical samples containing hPIV RNA to evaluate their performance and efficiency. Sequencing results demonstrated high-quality genome data and efficient amplification across various Ct values. As a result, 41 hPIV1 and 13 hPIV2 near-complete genome sequences were successfully obtained from clinical specimens collected in Saint Petersburg (Russia). Phylogenetic analysis of the HN gene sequences showed that Russian hPIV1 strains clustered into clades II and III, while hPIV2 strains were distributed between clusters G1a and G3. The whole-genome-based trees confirmed the same distribution of the strains. These findings highlight the potential of our primer panels and contribute to a better understanding of the molecular characteristics and phylogenetic diversity of circulating hPIV strains. Notably, this study presents the first evolutionary analysis of hPIVs in Russia.

## 1. Introduction

Human parainfluenza viruses (hPIVs) are a prominent etiological cause of acute respiratory tract infections (ARIs) [1]. hPIVs are most frequently associated with croup but can lead to a wide spectrum of respiratory illnesses. Infections with hPIVs are ranking second only to respiratory syncytial virus (RSV) as a cause of ALRI-associated hospitalization in children under the age of five years [2,3,4,5].

hPIVs are a group of spherical, enveloped, single-stranded RNA viruses that belong to the family *Paramyxoviridae* in the order *Mononegavirales* and have been divided into four distinct types, which fall into two genera *Respirovirus* (hPIV1 and hPIV3), and *Rubulavirus* (hPIV2 and hPIV4) [6]. They are restricted in their tropism to the respiratory tract epithelium [7]. Outbreaks of hPIV3 infections typically happen during late spring and summer, while peaks of hPIV1 and hPIV2 infections occur in late autumn and early winter [8,9].

The paramyxovirus genome is approximately 14.9–17.3 kb in length, encodes six common structural proteins, in the invariant order: the nucleocapsid protein (N), phosphoprotein (P), matrix (M), fusion (F), hemagglutinin–neuraminidase (HN), and the large protein (L). Of these, F and HN glycoproteins are membrane-associated proteins, while N, P, and L are associated with the nucleocapsid [10]. The major antigenic determinants responsible for infecting the host cells are the hemagglutinin–neuraminidase (HN) and fusion (F) proteins [11]. The hemagglutinin–neuraminidase facilitates virus attachment to target cells, while the fusion protein mediates fusion between the viral envelope and the cellular membrane upon attachment [12,13]. Besides these common proteins, each hPIV also encodes at least one additional nonessential protein. hPIV1 and hPIV3 produce short C proteins from alternate open-reading frames in the P gene [14], whereas hPIV2 produces a V protein through an mRNA-editing mechanism in the P gene rather than a completely separate open reading frame [15,16].

Hemagglutinin–neuraminidase and fusion proteins exhibited extensive antigenic variability, making them the preferred targets of molecular epidemiological studies, whereas the remaining structural proteins were reported to be antigenically well-conserved [17,18,19,20].

Among the four hPIV serotypes, the prevalence of hPIV1 and hPIV2 ranked second and third after hPIV3, respectively [21,22]. Despite hPIV3 being recognized as a primary viral cause of acute respiratory infections in infants and young children, infections with hPIV1 and hPIV2 have been associated with seasonal outbreaks in older children, with hPIV1 being more frequently detected in children over the age of five, while hPIV2 showed variable detection rates depending on the region and time of year [23].

Currently, the complete genome sequence information available for hPIV1 and hPIV2 in GenBank database remains limited in its geographic scope and completeness. Most sequences are biased toward certain countries and predominantly represent the HN gene. Additional sequence information regarding hPIVs is necessary to comprehensively understand their genetic diversity, evolution, and potential recombination events. This understanding is vital for thorough monitoring, prevention, and control of these pathogens.

Genomic surveillance of hPIVs is not carried out in Russia. To date, there is no whole-genome data available that would allow for detailed epidemiological and phylogenetic analysis of circulating strains. The most efficient method for sequencing viral genomes is amplicon sequencing. In this study, we aimed to develop primer panels for sequencing the whole genome of hPIV1 and hPIV2.

## 2. Results

### 2.1. Prevalence of hPIV

**Figure 1:**
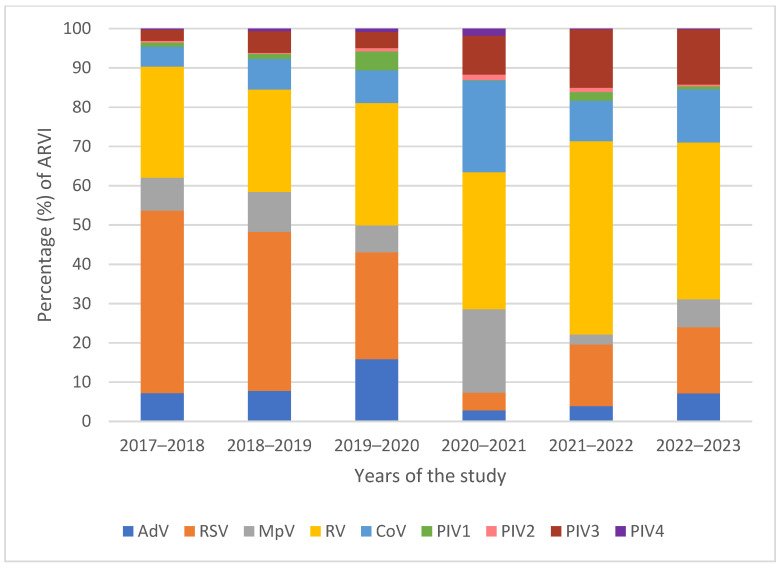
The percentage distribution of acute respiratory viral infections between 2017 and 2023. AdV—adenovirus, RSV—respiratory syncytial virus, MpV—metapneumovirus, RV—rhinovirus, CoV—seasonal coronavirus, PIV1—parainfluenza virus type 1, PIV2—parainfluenza virus type 2, PIV3—parainfluenza virus type 3, PIV4—parainfluenza virus type 4.

From October 2017 to September 2023, 1334 respiratory samples out of 10,408 ARVI (12.81%) were tested positive for hPIV (Figure 1). Most of the samples tested were positive for hPIV3 (79%), followed by hPIV1 (12%), hPIV2 (5%), and only 4% hPIV4 during this period. Mixed infections between parainfluenza subtypes were not observed, while co-infections with other respiratory viruses were more common (13.18%). Analysis of seasonal incidence in St. Petersburg showed that hPIV peaks annually in the spring period (47.3%), decreasing in the autumn months, accounting for approximately 8.84% of the total cases.

### 2.2. Primer Design and Genome Coverage

**Figure 2:**
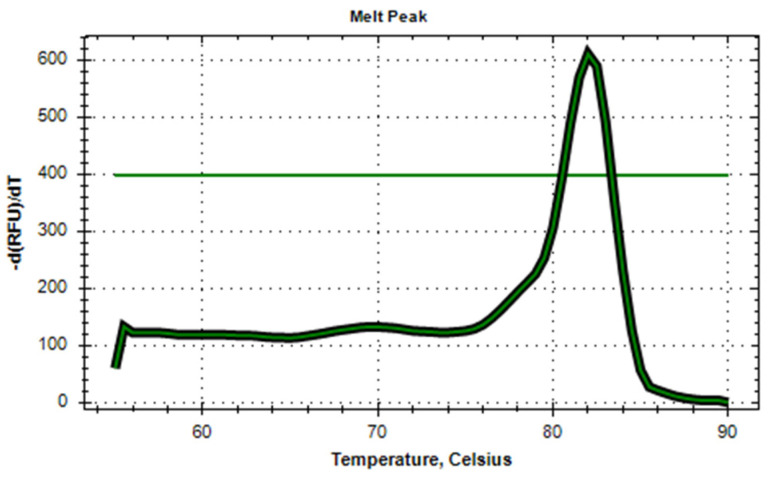
Example of a successfully amplified hPIV sample using the developed primer panel. The amplification was monitored using SYBR Green dye, demonstrating clear amplification curves with minimal non-specific signal.

**Figure 3:**
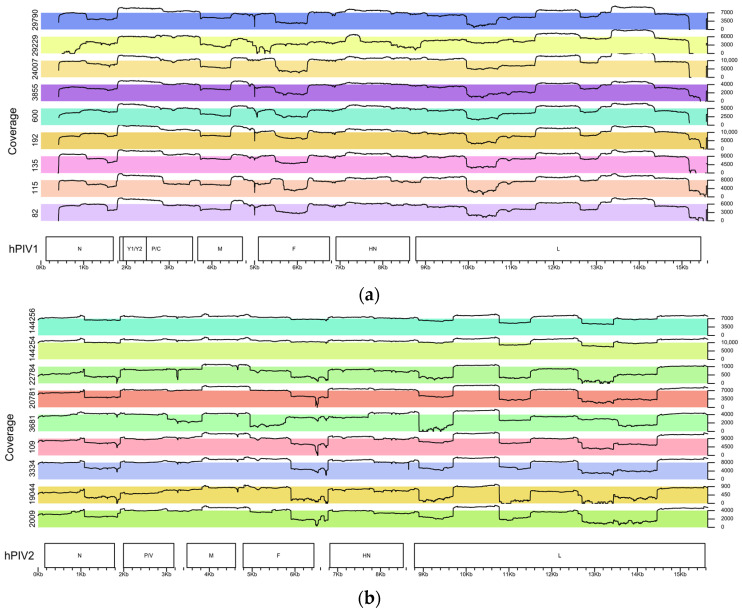
Coverage plots of hPIV genomes generated using the VizCoV script in the RStudio environment (https://github.com/LMV-NIC-St-Petersburg/VizCoV, accessed on 1 December 2024): (**a**) coverage plot representing nine hPIV1 samples after sequencing; (**b**) coverage plot representing nine hPIV2 samples after sequencing.

The amplification efficiency of the developed primer panels was first assessed using SYBR Green dye to monitor real-time PCR performance. An example of a successfully amplified sample is shown in Figure 2. The tiled amplicon set design resulted in uniform coverage of each of hPIV1 and hPIV2 consensus genomes sequences, resulting in high-quality and near-complete genomes with minimal gaps in sequencing (Figure 3). The average amplicon length is approximately 1 kb with an overlap of 200 bp between adjacent amplicons, ensuring robust coverage. Primers were targeted to the most conserved regions of the genome to maximize specificity and amplification success. Although a limited number of degenerate nucleotides were incorporated into the primer sequences, they were specifically included to enhance binding across diverse viral strains, without compromising amplification efficiency. With a data volume of 300 thousand reads per sample, the developed panels demonstrate a median coverage of 5793× of hPIV1 and 3980× of hPIV2 that allows for establishing a consensus genome sequence. During the study, 41 near-full-genome sequences of hPIV1 and 13 hPIV2 from Russia were obtained for the first time. While the amplification step was unsuccessful for a subset of samples due to potential RNA degradation, the primer panels developed in this study offer a valuable tool for genomic surveillance of ARVI pathogens.

### 2.3. Analytical Specificity

The primer panels for hPIV1 and hPIV2 demonstrated high specificity. Amplification signals were exclusively detected for hPIV1 and hPIV2 targets and their respective positive controls, confirming the panels’ intended specificity. No cross-reactivity observed with other hPIV types and or non-target respiratory pathogens (e.g., Inf A, Inf B, RV, RSV, CoV, MpV, and AdV) (see Table 1 for complete data). These findings confirm the high analytical specificity of the hPIV1 and hPIV2 primer panels, ensuring reliable detection and differentiation of each virus without interference from other respiratory pathogens.

### 2.4. Analytical Sensitivity

**Figure 4:**
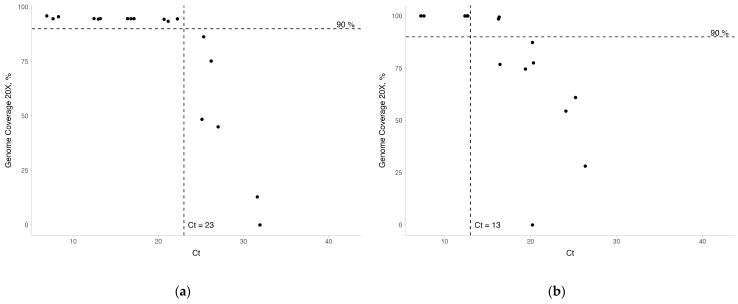
Scatterplots of assessing the primer panels sensitivity of hPIV1 and hPIV2 isolates’ dilution series by assessing sequencing parameters in relation to Ct values: (**a**,**b**) scatterplots of minimum coverage at 20× depth vs. Ct values for hPIV1 and hPIV2, respectively.

The analytical sensitivity of the primer panels was assessed for both hPIV1/Russia/SPE-RII-18746V/2023 and hPIV2/Russia/SPE-RII-17155V/2023 dilution series based on the Ct values obtained through real-time PCR and genome coverage achieved during sequencing. At a Ct value below 23, all hPIV1 samples achieved over 90% genome coverage at minimum 20× depth (Figure 4a). The genome coverage significantly declined for samples with Ct values exceeding 33, with no coverage observed for samples with Ct > 26. For hPIV2, genome coverage remained above 90% at Ct ≤ 24, indicating a narrower range for high-quality sequencing compared to hPIV1 (Figure 4b). These results indicate that the sensitivity of the primer panels is closely tied to Ct values, with Ct thresholds of 33 for hPIV1 and 24 for hPIV2 being critical for achieving high genome coverage during sequencing.

### 2.5. Evaluation of Primer Panels on Clinical Specimens

**Figure 5:**
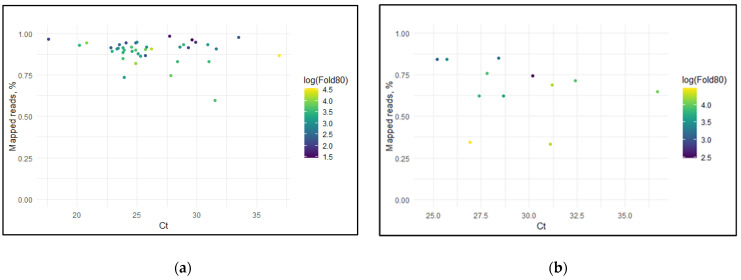

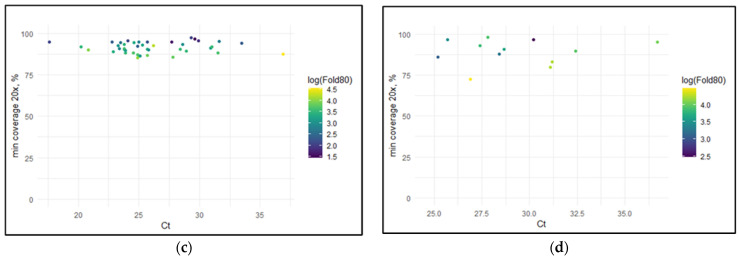
Scatterplots of the primer panels efficiency used for whole-genome sequencing of hPIV1 and hPIV2 by assessing sequencing parameters in relation to Ct values: (**a**,**b**) scatterplots of percentage mapped vs. Ct values for hPIV1 and hPIV2, respectively; (**c**,**d**) scatterplots of percentage min 20× vs. Ct values for hPIV1 and hPIV2, respectively. The color gradient represents the log(Fold80) value.

The performance of the developed panels was assessed by examining the following metrics: the percentage of reads mapped, the percentage of the genome covered at a minimum depth of 20× (% min 20×), and the Fold80 penalty as a metric of coverage uniformity, all in relation to the cycle threshold (Ct) values. The scatterplot of hPIV1 demonstrated a correlation between Ct values and percentage mapped of sequencing reads (Figure 5a). For samples with lower Ct values, the percentage mapped was consistently high, with most samples manifesting close to 100% of reads successfully aligned to the reference genome. However, whilst Ct values increased, we observe a reduction in mapping efficiency of samples, with the percentage of mapped reads dropping to around 75% for some samples. Furthermore, a similar trend was noticed for hPIV2 (Figure 5b), where samples with Ct values below 28 exhibited high mapping percentages (over 75%). However, for certain samples with Ct values greater than 31, the percentage of mapped reads drops to 50%, which confirms the direct Ct-values-mapped reads relation. Strikingly, two samples with similar Ct values (around 31) revealed a considerable difference in mapping efficiency, which may be explained by the variability in the quality of clinical samples and technical factors that could affect the performance.

Likewise, regarding the genome coverage at a minimum depth of 20×, both primer panels of hPIV1 and hPIV2 demonstrated remarkable performance for samples with Ct values below 28. The percentage of the genome covered at 20× was approximately 100% for low Ct—hPIV1 samples (Figure 5c). Similarly, for hPIV2, samples with Ct values below 18 showed nearly complete genome coverage at 20× depth (Figure 5d). However, in samples with higher Ct values, coverage of both hPIV genomes dropped significantly.

Across the four represented scatterplots, the right color gradient displaying log(Fold80)—a measure of the fold coverage required to cover 80% of the genome—showed that higher Ct samples require significantly more sequencing depth to obtain comprehensive genome coverage. This emphasizes the obstacles posed by high Ct values in terms of sequencing efficiency.

### 2.6. Robustness

Both the C35 and Greer strains showed positive amplification with the related developed primer panels and were successfully sequenced. This outcome demonstrates that the primer panels are robust and effective for whole-genome sequencing across phylogenetically distant hPIV1 and hPIV2 strains.

### 2.7. Phylogenetic Analysis

Phylogenetic analyses were performed using both the HN gene-coding sequence (cds) and the complete genome sequence to better understand the evolutionary relationships of hPIV1 and hPIV2.

#### 2.7.1. Phylogenetic Analysis of hPIV1

**Figure 6:**
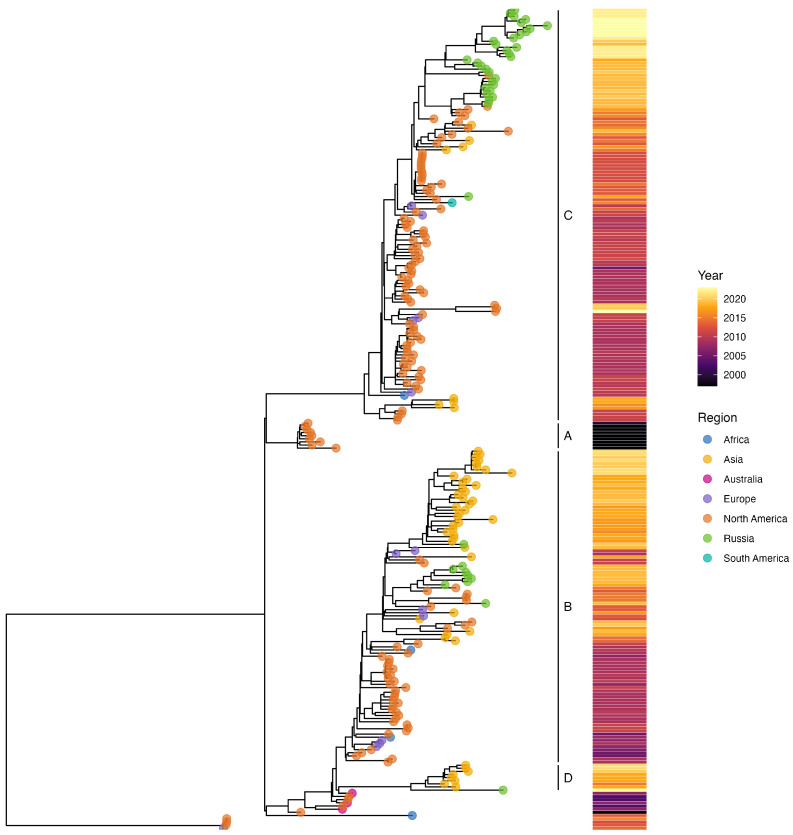
Phylogenetic tree based on whole-genome sequence of hPIV1. The phylogeny was reconstructed using the maximum likelihood (ML) method in the RAxML and visualized in R (ggtree). The four identified clades A, B, C and D are indicated. The Russian strains are labeled with green circles. For strain names, see Supplementary Figure S1.

The whole-genome-based ML phylogenetic tree of 224 hPIV1 (Supplementary Table S1a) sequences and 41 Russian sequences revealed four distinct clades: A, B, C, and D, as suggested previously by Zhu et al. [24]. The Russian hPIV1 strains were distributed across clades B, C, and D (Figure 6). Notably, clade D includes a single Russian strain (PP757765) clustered alongside previously reported strains from China, while nine others (PP886700, PP886678, PP886691, PP886688, PP886684, PP886681, PP886686, PP886677, and PP886685) were grouped in clade B. The majority of Russian strains (thirty one) were categorized into clade C.

Distinct substitution patterns were observed within each clade. In clade C, nearly all strains shared substitutions D256E in the P protein, V6I in the F protein, and A40T in the HN protein, except for strain PP886676, which exhibited unique mutations (H250Y in the P protein and S9N in the HN protein). A subgroup within clade C, including PP886683, PP886680, PP886695, PP886699, PP886692, PP886693, PP886698, PP886689, and PP886679, exhibited additional substitutions: G255E and T292A in the P protein, T279A in the M protein, and I8T in the HN protein. Furthermore, strain PP886696 was uniquely characterized by multiple substitutions, such as N151S in the N protein, F173L and I234V in the P protein, E307D and K546R in the F protein, and D462N and R520K in the HN protein. Other clade C strains, including PP886710, PP886706, PP886703, PP886694, PP886690, PP886707, PP886704, PP886705, PP886702, PP886711, PP757766, PP757764, PP757767, PP886709, PP886708, PP886697, PP886687, PP886701, PP886682, and PP886675, showed consistent substitutions: T244I and P309L in the P protein, N533S in the F protein, and K77R in the L protein. Clade B strains displayed shared substitutions such as Y34N in the HN protein and M339T in the L protein. Additionally, PP886700 exhibited unique changes, including T295A in the P protein and N333S and I509M in the F protein. Furthermore, clade D, comprising the Russian strain PP757765 along with Chinese strains, exhibited specific substitutions, such as A442T, N443H, and A517V in the N protein; S52N, S56N, K61R, P125L, and R196G in the P protein; L7F, V25I, V155I, and A214T in the F protein; and V22A, G31R, M66I, N355D, H385R, F466Y, K468E, K514E, and G524E in the HN protein. The substitutions were identified and visualized using Treesub and ggtree.

**Figure 7:**
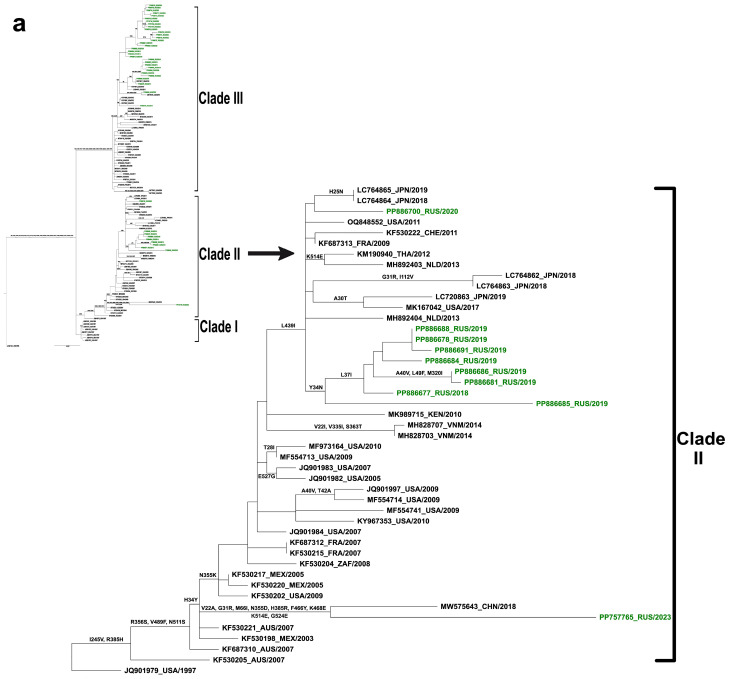

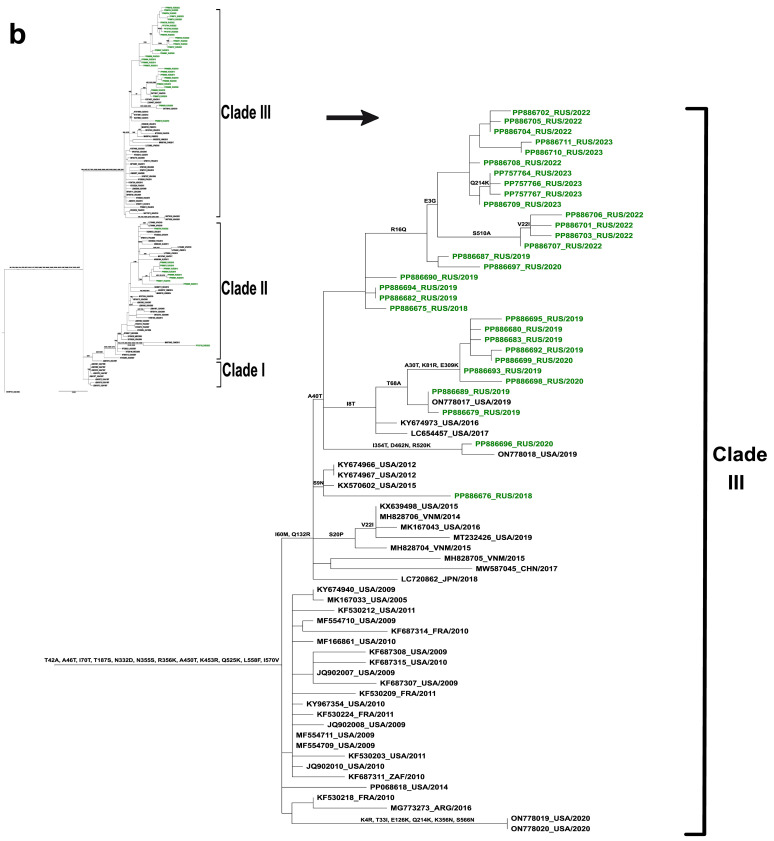
Phylogenetic analysis of hPIV1 based on HN gene constructed using a 1718 nucleotide sequence corresponding to nucleotide 6847–8740 in the Washington_1964 strain (GenBank accession no: NC_003461). The phylogeny of 125 HN gene sequences was analyzed using the maximum likelihood (ML) method in the RAxML and TreeSub programs. The country codes and year of collection are shown. The Russian strains obtained in this study are colored in green: (**a**) Clade II; (**b**) Clade III. The two main clades have been demonstrated separately for visibility reasons.

The HN-based phylogenetic analysis further refined these findings, aligning the Russian hPIV1 strains with 84 global sequences from 13 countries: USA, Japan, Vietnam, China, Kenya, Argentina, Thailand, Mexico, France, South Africa, Australia, Switzerland, and The Netherlands (See detailed information in Supplementary Table S3a) (CDS region; 1728 nt). Only GenBank available sequences obtained from wild-type hPIV1 viruses and have no gaps in coverage of the open reading frames in the HN gene are included. The HN gene phylogenetic tree grouped 125 hPIV1 genome sequences into three main branches, referred to as Clades I, II, and III (Figure 7a,b). Among these, clade I, which exclusively incorporates strains from the United States, had been in circulation in the late 1990s, while the two major clades (II and III) are currently prevalent and encompass circulating strains during the time of this study. Our strains were found to co-circulate within the two phylogenetic clades (II and III); however, the majority of them have been grouped within clade III. Eight Russian samples (PP886688, PP886678, PP886691, PP886684, PP886686, PP886681, PP886677, PP886685) isolated in the seasons 2017/18, 2018/19, and 2019/20 were grouped together in clade II (Figure 7a), while one sample (PP886700) was shown to be more closely related to Japanese strains (LC764865 and LC764864). Noteworthy, the strain hPIV1/Russia/SPE-RII-5007S/2023 (PP757765) formed a separate branch in clade II along with virus MW575643 isolated from China in 2018. Clade III was shown to be more diverse, as it includes the thirty-one remaining Russian strains that have been isolated during the studied epidemiological seasons (Figure 7b). All the strains were assembled together close to the top end of the phylogenetic tree, interspersing with strains isolated from the United States in 2020 (ON778017 and ON778018). One strain hPIV1/Russia/SPE-RII-635S/2018 (PP886676) circulation was demonstrated with the most related strains KY674966 (2012), KY674967 (2012), and KX570602 (2015), isolated from the USA, as well.

For each group, characteristic sets of amino acid substitutions in the HN glycoprotein were identified. Analysis has shown eleven common substitutions related to clade III (T42A, A46T, I70T, T187S, N332D, N355S, R356K, K453R, Q525K, L558F, I570V) and nineteen common substitutions related to clade II (N8I, T45A, V46A, F49L, M76V, R131K, S151T, I335V, D349N, T358P, N443K, E453K, K448N, Q461P, Y466F, R468K, E514K, V524G, A553T).

#### 2.7.2. Phylogenetic Analysis of hPIV2

**Figure 8:**
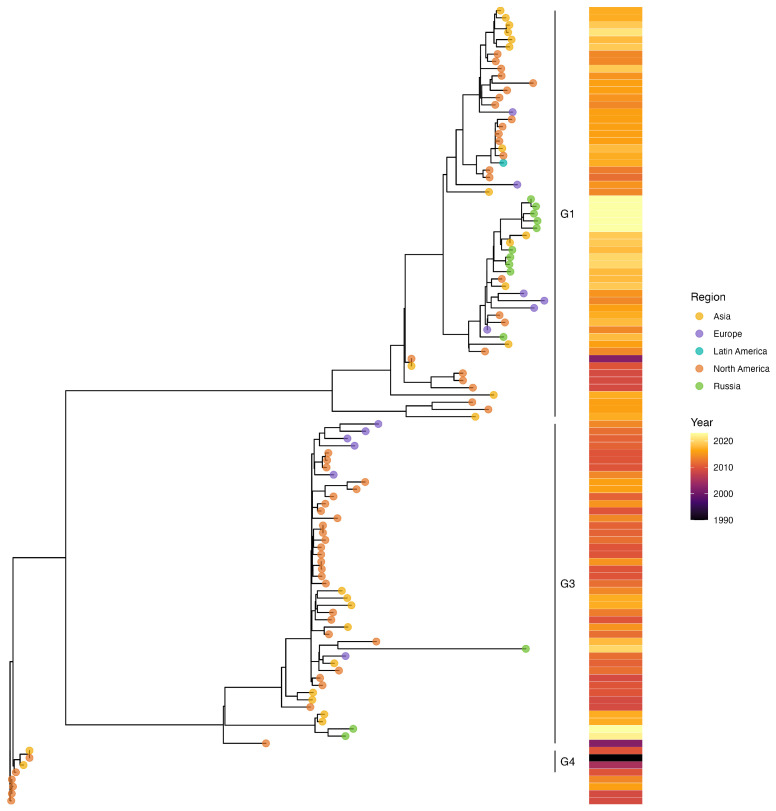
Phylogenetic tree based on whole-genome sequence of hPIV2. The phylogeny was reconstructed using the maximum likelihood (ML) method in the RAxML and visualized in R (ggtree). The Russian strains are labeled with green circles. For strain names, see Supplementary Figure S2.

The ML phylogenetic tree of the whole-genome sequences of 97 global hPIV2 strains (Supplementary Table S1a), along with 13 Russian sequences, identified four distinct clades: G1 to G4. The Russian strains were distributed across two clades, G1 and G3 (Figure 8), with three strains (PP886717, PP886721, PP886718) belonging to G3 and the remaining 10 strains (PP886719, PP886720, PP737253, PP886722, PP737254, PP886712, PP886716, PP886715, PP886714, and PP886713) grouped within G1 (subclade G1a).

The subgroup of G1a strains, including the Russian strains, was characterized by the following specific amino acid substitutions: P439S in the N protein, D469E and A524T in the F protein, E254D in the HN protein, and I329V and L2239P in the L protein. Among the three Russian strains classified within the G3 clade, two formed a distinct subgroup characterized by specific substitutions identified through ancestor strain reconstruction using TreeSub (powered by PAML). These substitutions included L485P in the N protein, I93T in the P protein, N247K and K289R in the F protein, seven changes in the HN protein (T117S, K139N, E254D, P319S, N322K, A348T, N381S), and three in the L protein (I329V, T2181I, I2245M). One strain, hPiv2/Russia/SPE-RII-3410S/2020 (PP886717), classified within the G3 clade, stands apart from the other Russian sequences and was subsequently identified as a recombinant (see details below).

**Figure 9:**
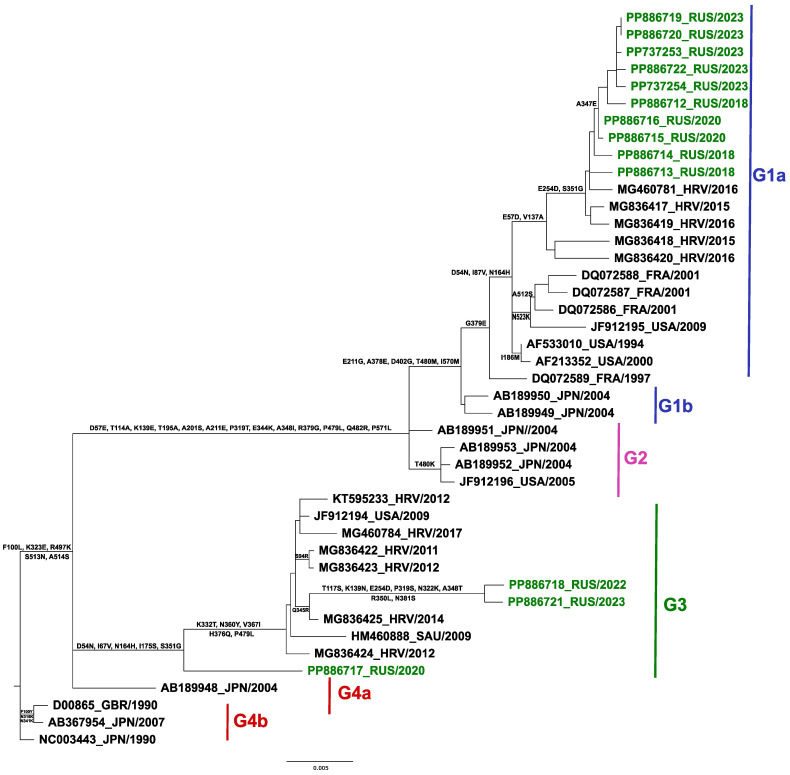
Phylogenetic analysis of hPIV2 based on HN gene constructed using a 1715 nucleotide sequence corresponding to nucleotide 6817–8532 in the Japan_1990 strain (GenBank accession no: NC_003443). The phylogeny of 43 HN gene sequences was analyzed using the maximum likelihood (ML) method in the RAxML and TreeSub programs. The country codes and year of collection are shown. The Russian strains obtained in this study are colored in green.

Phylogenetic analysis was conducted based on the HN gene sequences of 30 hPIV2 strains available in GenBank (Supplementary Table S3b). This analysis confirmed a similar distribution, with the Russian sequences clustering into two groups, G1a and G3 (Figure 9). Notably, these clusters demonstrated close phylogenetic relationships to Croatian strains (MG460781_HRV/2016, MG836423_HRV/2012, MG836424_HRV/2012, and MG836425_HRV/2014). Analysis of the inferred amino acid sequence alignment revealed 5 common positions of potential substitutions between the Russian isolate hPIV2/Russia/SPE-RII-3410S/2020 (PP886717) and the other G3-grouping strains (D54N, I67V, N164H, I175S, S351G). However, eight additional variations were characterized only in hPIV2/Russia/SPE-RII-2009S/2022 (PP886718) and hPIV2/Russia/SPE-RII-20781S/2023 (PP886721) strains (T117S, K139N, E254D, P319S, N322K, A348T, R350L, and N381S). While all strains of the G1a cluster share three common variations (D54N, I87V, N164H), the remaining Russian strains include three more (E254D, S351G, A347E).

The majority of the observed substitutions (hPIV1/*n* = 18, hPIV2/*n* = 24) altered the amino acid properties related to charge, polarity, and/or hydropathy.

### 2.8. Potential Glycosylation Sites Analysis

#### 2.8.1. Potential Glycosylation Sites Analysis of hPIV1

Regarding the HN protein of hPIV1, eight N-glycosylation sites were predicted at amino acid positions 19, 77, 173, 277, 361, 499, 504 and 511. Six of them are conserved in all analyzed viruses (19, 173, 277, 361, 499 and 504), while nine strains (PP886677, PP886678, PP886681, PP886684, PP886685, PP886686, PP886688, PP886691, and PP886700) lack the N-glycosylation site 511 (N511S). Additionally, glycosylation of aa 77 is predicted only in seven samples (PP886680, PP886683, PP886692, PP886693, PP886695, PP886698, and PP886699). Of interest, the reference N-glycosylation site at position 8 (NC_003461_USA_1964) is absent in all of our analyzed sequences. Since the regions spanning residues 191–341 to 457–504 are the most conserved parts of the HN protein [25,26], it is likely that glycosylation within this domain plays a crucial role in maintaining the structural integrity, potentially supporting its proper folding or aiding in the assembly of HN monomers into functional tetramers [26]. These modifications may also enhance viral pathogenicity by masking key antigenic sites from neutralizing antibodies, thereby promoting immune evasion and viral growth.

#### 2.8.2. Potential Glycosylation Sites Analysis of hPIV2

Eight potential N-linked glycosylation sites with a score above 0.5 were identified in the HN protein of hPIV2 sequences (amino acid positions 6, 272, 284, 316, 335, 341, 501, and 517). An additional potential site was detected at position 47 specifically in the PP886712 strain. It is noteworthy, however, that the glycosylation site at position 316 overlaps with a recognized antigenic region [27], suggesting that glycosylation at this position could impact viral pathogenicity and immune recognition.

### 2.9. Recombination Analysis

One hPIV1 strain, hPiv1/Russia/SPE-RII-6452S/2023, classified within clade C, was identified as having undergone a recombination event. The potential major parent was hPiv1/Russia/SPE-RII-1874S/2023 (clade C). The potential minor parent was hPiv1/Russia/SPE-RII-5007S/2023 (clade D). The recombination event was localized to the non-coding F gene region, spanning nucleotide positions 4966 to 5100.

A recombinant strain of hPIV2, designated hPiv2/Russia/SPE-RII-3410S/2020 (PP886717) and classified within clade G3, was identified. Analysis suggests the major parental strain was the US strain 2018_9783 (MT118675), also belonging to clade G3, while the minor parental strain was hPiv2/Russia/SPE-RII-109S/2018 (PP886714), classified within clade G1. The recombination event occurred in the L gene region, spanning nucleotide positions 10,762 to 12,660.

## 3. Discussion

Human parainfluenza viruses (hPIVs) are an important cause of respiratory illness in children and adults with a wide range of clinical manifestations [6]. Both hPIV1 and hPIV2 are associated with croup in children and can lead to outbreaks of acute respiratory disease, contributing to considerable morbidity rates [28,29]. Currently, there is a dearth of whole-genome sequence data that would allow for detailed epidemiological and phylogenetic analysis of circulating strains in Russia.

In this study, we presented a robust amplicon-based sequencing pipeline for whole-genome sequencing of hPIV1 and hPIV2 to evaluate the genetic variability of circulating Russian strains. The depth of coverage obtained across all sequenced genomes was consistently high. The zero-covered positions of the genomes were less than 5% for hPIV1 and less than 1% for hPIV2. The performance of the designed primer panels was evaluated through a comparative analysis of Ct values, percentage of reads mapped to the genome, and the percentage of genome coverage at a minimum depth of 20×. For hPIV1, the percentage of mapped reads remained high between Ct values 20 and 35, demonstrating the efficiency of the primer panel across this range. Even at higher Ct values, the percentage of mapped reads remained above 75%. A similar trend was observed for hPIV2, where Ct values ranged from 25 to 35, and despite the greater variability in Ct values, the percentage of mapped reads remained relatively stable. In both cases, the panels showed good performance in maintaining a high percentage of mapped reads even as Ct increased. For both hPIV1 and hPIV2, the genome coverage at a minimum depth of 20× was consistently high across a range of Ct values. All these metrics underscore the quality of the sequencing results and the high efficiency of the panels in amplifying viral genomes. By developing these novel primer panels for complete genome sequencing, we conducted the first phylogenetic analysis of these viruses in Russia.

Forty-one hPIV1 and thirteen hPIV2 near-complete genome sequences were obtained directly from clinical nasal swab specimens from 2017 to 2023 collected in Saint Petersburg. These data were combined with hPIV1 and hPIV2 gene sequences publicly available in GenBank to perform a comprehensive phylogenetic analysis.

For hPIV1, the whole-genome-based ML phylogenetic tree revealed four clades (A–D), with Russian strains distributed across clades B, C, and D. Most Russian sequences were clustered within clade C, while nine were grouped in clade B, and a single strain (PP757765) in clade D clustered alongside strains previously reported in China [24].

Previous evolutionary studies of hPIV1, based on the HN gene sequence, identified three genetic clades, two major and one minor [30,31]. The phylogenetic analysis based on the HN-gene-coding sequence corroborated the findings from the whole-genome analysis, allocating Russian hPIV1 strains into two distinct branches, corresponding to clades II and III, with the majority clustering in clade III. These samples show the closest genetic relationship to virus sequences from China, Japan, and the USA [32,33,34].

Regarding hPIV2, earlier phylogenetic analyses that used the coding region of the HN gene identified four genetic clusters (G1–G4) [35,36]. The 13 Russian hPIV2 sequences from this study were distributed between two distinct clusters, G1a and G3, both of which exhibited close genetic proximity to circulating strains from Croatia [37]. The same phylogenetic distribution was demonstrated by the whole-genome-based ML phylogenetic tree of hPIV2, as well. By integrating whole-genome- and HN-gene-based phylogenetic analyses, this study provides a comprehensive view of the genetic diversity of Russian hPIV1 and hPIV2 strains within the global context.

Overall, the arrangement of both hPIV1 and hPIV2 Russian strains in the HN gene trees and whole-genome trees does not correlate with epidemic seasons, suggesting that strains from both clades circulated simultaneously in Russia. Moreover, the resulting phylogeny indicates that the distribution of hPIV1 and hPIV2 strains is not linked to geographical location but rather depends on the strains circulating temporally.

Recombination events play crucial roles in shaping the genetic diversity and evolutionary trajectory of RNA viruses, enabling them to adapt to new hosts and evade immune responses [38]. In this study, we investigated the potential recombination events of hPIV1 and hPIV2 strains to better understand their evolutionary dynamics. The long non-coding region of the hPIV1 F gene contains cis-acting elements that influence transcriptional termination [39]. We described clade C/D recombinants. Previous studies have demonstrated that the hPIV1 F gene may harbor recombination breakpoints, as evidenced by a recombination event identified between nucleotide positions 4497 and 5543 in HPIV1/CHN/YC17073/2017 (YC17073), a clade B/C recombinant [24]. The functional significance of these recombination events remains unclear; however, it is speculated that they could influence the production of M-F read through mRNAs, which have been previously described in hPIV1-infected cells [40].

The comprehensive genomic data obtained will enable us to understand hPIV genetic diversity and epidemiology, ultimately contributing to better monitoring and control approaches for these respiratory pathogens. Further studies should be conducted to obtain detailed and accurate genetic characterization of circulating hPIV strains, which is critical for providing insights into the global burden of respiratory illnesses caused by these viruses.

## 4. Materials and Methods

### 4.1. Ethics Statement

Samples used in this study were collected as part of approved ongoing hospital surveillance conducted by the Smorodintsev Research Institute of Influenza. The GIHSN (Global Influenza Hospital Surveillance Network) study was approved by the local ethics committee (Smorodintsev Research Institute of Influenza Local Ethics Committee). Written informed consent was obtained from all subjects in accordance with the order of the Ministry of Health of the Russian Federation of 21 July 2015 #474n.

### 4.2. Clinical Specimens

Collection and biobanking of oro- and nasopharyngeal swabs obtained from hospitalized adults and children aged from 1 month to 5 years, admitted as part of hospital surveillance of influenza and acute respiratory viral infections project (GIHSN) between the years 2017/18 and 2022/23. The samples were received from three hospitals: Clinical Infectious Disease Hospital named after S.P. Botkin, City Children’s Hospital of St. Olga, and St. Petersburg State City Children’s Clinical Hospital No. 5 named after N.F. Filatov, Saint Petersburg, Russia. Materials were transferred immediately to the Smorodintsev Research Institute of Influenza, Saint Petersburg. All specimens were registered and frozen at −70 °C.

### 4.3. RNA Extraction and RT-PCR

Isolation of RNA was performed on Biolabmix (Biolabmix^®^, Novosibirsk, Russia) columns RU-250 and on magnetic particles NAmagp100 using AutoPure-96 (Allsheng, Hangzhou, China) automatic station. RNA was suspended in 60 μL of elution buffer and stored at −70 °C. Detection of respiratory pathogens was carried out via a real-time PCR using commercial kits “AmpliSens^®^ ARVI-screen-FL” assay (Central Research Institute of Epidemiology, Russia), in accordance with the manufacturer’s instructions. This screening process targeted multiple respiratory viruses, including adenoviruses (AdVs), respiratory syncytial viruses (RSVs), metapneumoviruses (MpVs), rhinoviruses (RVs), parainfluenza viruses (PIVs), and coronaviruses (CoVs). In the “AmpliSens^®^ ARVI-screen-FL” assay, according to the manufacturer’s instructions, there is no fluorescence signal detection within the first 10 cycles, resulting in all Ct values being artificially lower than the actual values. To improve clarity, we adjusted the Ct values by adding 10 (Ct + 10). hPIV Samples with Ct value less than 35 were included in this study. Double-positive samples were excluded from the analysis.

### 4.4. Design of Multiplex Primer Panels

**Figure 10:**
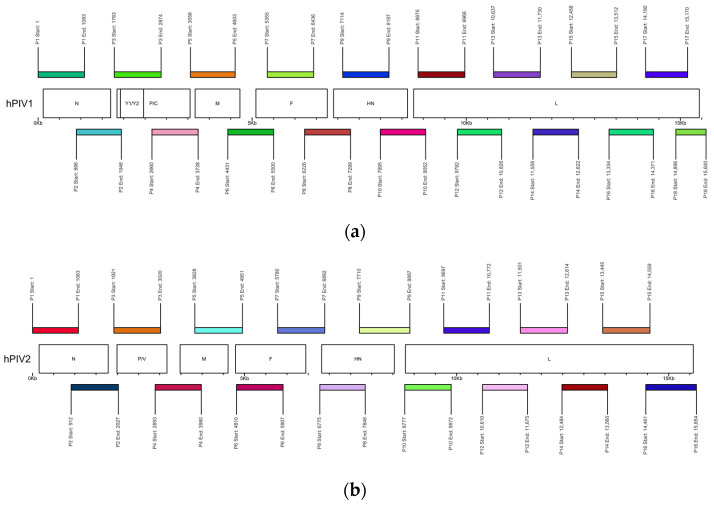
Diagrams illustrating the binding positions of primers along the genomes of hPIV, made using the VizCoV script in the RStudio environment version 2023.09.1+494 (https://github.com/LMV-NIC-St-Petersburg/VizCoV, accessed on 1 December 2024): (**a**) schematic representation of primer positions mapped across the genome of hPIV1; (**b**) schematic representation of primer positions mapped across the genome of hPIV2.

Two sets of a total of 18 (hPIV1) and 16 primer pairs (hPIV2) were designed using PrimalScheme tool (https://primalscheme.com, accessed on 1 December 2024) to cover the whole viral genome (Table 2a,b). These primer designs were based on consensus sequence genomes obtained from multiple alignments of complete 227 hPIV1 and 97 hPIV2 genomes available in NCBI (See detailed information in Supplementary Table S1a,b). Each primer pair covers approximately 1 kb of the genome with about 200 bp overlap of amplicons. Figure 10a,b represent schematic illustrations of the arrangement of the designed set of primers along hPIV1 and hPIV2 genomes.

### 4.5. Whole-Genome Amplification

The developed panels of primers were tested on clinical samples containing RNA of hPIVs according to screening data using the AmpliSens “ARVI-screen” reagent kit. Samples with a threshold cycle value (Ct) < 35 were selected for testing. Ct values were used as an indicator of viral RNA abundance (viral load) in samples. In total, 110 hPIV1 and 37 hPIV2 samples were amplified using the designed primer panels. For each parainfluenza sample, two separate multiplexed PCR reactions (pools 1 and 2) were generated using a one-step method kit from Biolabmix BioMaster RT-PCR Premium (2×) (Biolabmix^®^, Novosibirsk, Russia) as directed by the manufacturer. RT-PCR was performed under the following conditions: reverse transcription step at 45 °C for 1 h, pre-denaturation at 93 °C for 5 min, denaturation at 93 °C for 10 s, annealing at 54 °C for 30 s, elongation at 68 °C for 4 min (44 amplification cycles), and final elongation at 68 °C for 7 min. SYBR Green was used to effectively evaluate the success of amplification. The positively amplified PCR products in pool 1 and 2 reactions for the same samples were pooled together and used for NGS library preparation.

### 4.6. hPIV Sequencing

The preparation of DNA libraries for sequencing was carried out in several steps using a free –PCR commercial kit MGIEasy Fast PCR-free FS DNA Library Prep Set (MGI Tech, Shenzhen, China) according to the manufacturer’s instructions with minor optimizing adjustments, which include longer fragmentation time (25 min versus 7, 5 min in original protocol), altered beads to DNA ratio for cleanup of fragmentation product (1.2× versus 0.8× in original protocol), and longer adapter ligation time (17 min versus 10 min in original protocol). The original protocol is suitable for PE100 and PE150 modes of sequencing, and since we are using SE100 mode, we are forced to alternate protocols to receive shorter DNA libraries (aiming to 300 bp length of library; 100–150 bp insert length and 132 bp adapter length). Whole-genome sequencing was performed using MGI DNBSEQ G-400 platform (MGI Tech, Shenzhen, China) in single-end read mode with a length of 100 base pairs. Additionally, part of the samples was processed using Illumina DNA Prep kit (Illumina, San Diego, CA, USA) and sequenced on Illumina NextSeq 2000 platform in PE300 paired-end read mode.

### 4.7. Bioinformatics Data Analysis

Consensus genome assembly was performed using BWA v. 0.7.17, Samtools v. 1.19.2, and Ivar v.1.4.2, along with custom Python scripts. Multiple alignment of nucleotide sequences with sequences from the GenBank database was performed using MAFFT software [41]. Phylogenetic analysis was performed using RAxML [42] and TreeSub (https://github.com/tamuri/treesub, accessed on 1 December 2024) programs using the maximum likelihood (ML, GTR+G) method. FigTree tool v1.4.4 (http://tree.bio.ed.ac.uk/software/figtree/, accessed on 1 December 2024) used for visualization of the generated phylogenetic tree. The amino acid sequences were generated by translating partial nucleotide sequences of the HN gene using the standard genetic code in MEGA11 (https://www.megasoftware.net/, accessed on 1 December 2024). These resultant amino acid sequences were then compared with those of previously documented strains from each cluster, with the Washington/1964 prototype strain (GenBank accession number: NC_003461) and Japan/1990 prototype strain (GenBank accession number: NC_003443) used as the references for hPIV1 and hPIV2, respectively. Mutations at every codon position were visualized using Unipro UGENE (https://ugene.net/, accessed on 1 December 2024) and MEGA11. Potentially N-glycosylated sites (Asn/X/Ser/Thr, where X is any amino acid other than proline) in hemagglutinin neuraminidase protein were predicted using the NetNGlyc server (https://services.healthtech.dtu.dk/service.php?NetNGlyc-1.0, accessed on 1 December 2024). Sites with threshold scores above 0.5 were categorized as glycosylated.

### 4.8. Specificity Test

The specificity of each developed primer panel was evaluated using clinical samples and positive controls for human parainfluenza viruses (hPIVs) and other respiratory pathogens. Clinical samples were obtained from the primary respiratory specimen biobank of the Smorodintsev Research Institute of Influenza (RII) and included positive samples for hPIV1, hPIV2, hPIV3, hPIV4, and other respiratory viruses: influenza A (Inf A), influenza B (Inf B), rhinovirus (RV), respiratory syncytial virus (RSV), coronavirus (CoV), metapneumovirus (MpV), and adenovirus (AdV). Additionally, hPIV1 and hPIV2 isolates were used as positive controls: hPIV1/Russia/SPE-RII-18746V/2023, Human parainfluenza 1 Virus, strain C35 (ATCC), hPIV2/Russia/SPE-RII-17155V/2023, and Human parainfluenza Virus 2, Strain: Greer (ATCC). Human parainfluenza 1 virus, strain C35, ATCC-VR-94, FR-295 and Human parainfluenza virus 2, (HPIV-2), strain Greer, ATCC-VR-92, FR-296 were obtained through the International Reagent Resource, Influenza Division, WHO Collaborating Center for Surveillance, Epidemiology and Control of Influenza, Centers for Disease Control and Prevention, Atlanta, GA, USA. The amplification reactions were conducted under identical conditions using each hPIV primer panel, and specificity was determined by the absence of fluorescence signals for non-target viral RNA.

### 4.9. Analytical Sensitivity

To determine the analytical sensitivity, we performed 10-fold serial dilutions (log10 dilution) on alpha-MEM medium containing hPIV1/Russia/SPE-RII-18746V/2023 and hPIV2/Russia/SPE-RII-17155V/2023 isolates, each with an infectious titer of 64 in the undiluted stock. The dilutions were tested in three replicates to estimate the Ct cut-off value of each primer set. Samples at each dilution level were first tested by real-time PCR using commercial “AmpliSens^®^ ARVI-screen-FL” assay. Furthermore, whole-genome amplification was conducted using the developed primer panels and verified using a melting-curve analysis, followed by sequencing on Illumina NextSeq platform. Analytical sensitivity was assessed depending on genome coverage metric: Genome Coverage ≥20× (The percentage of the genome covered by reads with at least 20-fold depth. The sequencing and genome coverage results were plotted against Ct values to analyze the sensitivity thresholds.

### 4.10. Robustness Test

To test the robustness of the primer panels, Human parainfluenza 1 Virus, strain C35 (ATCC), and Human parainfluenza Virus 2, strain Greer (ATCC), were selected for evaluation. These strains were chosen specifically for their phylogenetic distance from the Russian samples to confirm the primer panels’ efficacy across diverse viral lineages.

### 4.11. Recombination Analysis

Recombination analysis of hPIV1 and hPIV2 near-whole-genome sequences was performed using multiple algorithms within the RDP v.5.64 software suite [43], including RDP, GENECONV, BootScan, MaxChi, Chimaera, SiScan, 3Seq, LARD, and Phylpro. To ensure the reliability of the findings, recombination events were deemed valid only if detected by at least three of these algorithms.

### 4.12. Data Availability

All sequences of hPIV1 and hPIV2 described in this study have been deposited in GenBank under the Accession Nos. PP7577674-PP757767, PP886675-PP886722 (See detailed information in Supplementary Table S2a,b).

## 5. Patents

Patent pending. Application No. 2024120035 (Russian Patent Office).

## Figures and Tables

**Table 1:** Analytical specificity of hPIV1 and hPIV2 primer panels.

Sample	hPIV1 Primer Panel	hPIV2 Primer Panel
hPIV1	+	−
hPIV2	−	+
hPIV3	−	−
hPIV4	−	−
Inf A	−	−
Inf B	−	−
RV	−	−
RSV	−	−
CoV	−	−
MpV	−	−
AdV	−	−
hPIV1/Russia/SPE-RII-18746V/2023	+	−
hPIV2/Russia/SPE-RII-17155V/2023	−	+
Human parainfluenza 1 Virus, strain C35 (ATCC)	+	−
Human parainfluenza Virus 2, Strain: Greer (ATCC)	−	+

+ Amplification signal was detected, − no amplification signal was detected.

**Table 2:** (**a**) The designed primer panels for whole-genome amplification of hPIV1. (**b**) The designed primer panels for whole-genome amplification of hPIV2.

**(a)**					
**Name**	**Sequence**	5′-Position *	**Length**	**Tm**	**GC**
hPIV1-F1	ACCAAACAAGAGRAAAAACTTGTTT	1	25	60.3	32
hPIV1-R1	CATGAACTGGGTCTCTGAGTATACATATA	1083	29	60.1	37.9
hPIV1-F2	TTACATAAGAGATGCAGGATTAGCATC	896	27	61	37
hPIV1-R2	CGACGTCRAGGAGTCCGATG	1946	20	61	60
hPIV1-F3	CAGCATACACGAAACCAACCTT	1783	22	59.6	45.5
hPIV1-R3	AATTCATGTTGTGATTTCATTACACC	2874	26	59.6	30.8
hPIV1-F4	CCGTYAAAGAACGAAGAGCC	2660	20	59.8	55
hPIV1-R4	TGAGRGGGAGAGGTTCTACTGT	3738	22	59.4	54.5
hPIV1-F5	TCYACAATTTCAACCAGCAATC	3558	22	60.1	40.9
hPIV1-R5	GCTGCCCAGATGACTAGATTCAT	4603	23	60.2	47.8
hPIV1-F6	ATTGAGAAGATGAAGYTAATATTCTCTCT	4431	29	59.6	31
hPIV1-R6	GCTAGTGCAATTCCYGCAGTTATC	5500	24	60.5	45.8
hPIV1-F7	GATCTTCAGGAATCCCTGATAACA	5355	24	59.4	41.7
hPIV1-R7	GGACCCACTTTGATGATTTGATT	6436	23	59.8	39.1
hPIV1-F8	CATTCATAAATGGTGGTGTRGTAGCTA	6226	27	59.6	37
hPIV1-R8	CCYGTTTGTTGCATGACTTCTCTAT	7299	25	59.9	40
hPIV1-F9	TGACAGTATCCTCCGTGAACGA	7114	22	60.4	50
hPIV1-R9	GAGTGCCAACCTGARGATCTAGTATATA	8197	28	59.7	39.3
hPIV1-F10	TGCAATGATGCTCTTAAGATAACTTG	7995	26	60.5	34.6
hPIV1-R10	AAAATCGGATAAGGTTCAAAAGTGTA	9052	26	60.5	30.8
hPIV1-F11	TGCTAGAYATCAATCAACCTTATGATT	8875	27	61	33.3
hPIV1-R11	TTGCATGACACTCATATAGTGTCTTTAGTAT	9966	31	61.4	32.3
hPIV1-F12	TCATTGATAAAGATTTTCAGAGAGACAT	9792	28	59.8	28.6
hPIV1-R12	CTCATTACATCTTTGACCAAACAATG	10,826	26	60.3	34.6
hPIV1-F13	AAATATGAATAAGTGCAATTCAAATGG	10,673	27	60.9	25.9
hPIV1-R13	CTGGTTCTTGATTCATCACACGA	11,730	23	60.1	43.5
hPIV1-F14	GCAATATTGATYCCRGCTAATATAGG	11,559	26	60.6	38.5
hPIV1-R14	GTCGATACAGGAGTGAGTAACTTCAAA	12,621	27	60.7	40.7
hPIV1-F15	TGTTAAGAACTTAAGCAAGCCGG	12,458	23	61.1	43.5
hPIV1-R15	CACGCATGAAAAGATCAACAGAGTA	13,512	25	61.5	40
hPIV1-F16	AGACACATCACATGCAGTCYTAAAAGT	13,334	27	61	37
hPIV1-R16	AAAACCTTGACCCTTTGACATAAACT	14,371	26	61.5	34.6
hPIV1-F17	GCRGGTGCAATGCTGTCTTGT	14,190	21	61	52.4
hPIV1-R17	AATCTTGTAGACATAGACAATGCTATCCA	15,172	29	61.8	34.5
hPIV1-F18	AAGCATTACAAATCTTCGGATTTGA	14,896	25	61.8	32
hPIV1-R18	ACCAGACAAGAGTTTAAGAAATATCGATAT	15,600	30	61.3	30
(**b**)					
**Name**	**Sequence**	5′-Position **	**Length**	**Tm**	**GC**
hPIV2-F1	ACCAAGGGGAGAATTAGATGGC	1	22	61.4	50
hPIV2-R1	ATGGGTCCTAGACTCTGATARTGTAGC	1083	27	61.2	44.4
hPIV2-F2	CYATGGTGGGAGACATTGGCA	912	21	60.6	52.4
hPIV2-R2	GCTCAGTGGTGTATGTTGGTTCC	2027	23	61	52.2
hPIV2-F3	AADCATAGGCCCGGACGG	1921	18	60.5	51.1
hPIV2-R3	GGATTCRGGCTTTCGTGTGATC	3020	22	61.1	50
hPIV2-F4	YTCCAGTAGTAATTGCYGGTCC	2893	22	61	50
hPIV2-R4	ATAYTCAAATCCAGCTTGTAGCTTTG	3980	26	61.1	38.5
hPIV2-F5	AAGACATCAAGCCAGAGRGAGGA	3828	23	59.9	47.8
hPIV2-R5	AATAAAGCTAGCRCCACCATCAGT	4951	24	59.5	41.7
hPIV2-F6	AATGATAGTATGCATCTTTGTTATGTACACT	4810	30	59.8	30
hPIV2-R6	CGCTTRAGAAAGTTCCCGATAATA	5907	24	59.8	37.5
hPIV2-F7	GTGACACCRAACTCTGTATTYTGTAG	5780	26	59.6	42
hPIV2-R7	GGCAGTTCGGAAAATGATTCTA	6892	22	59	40.9
hPIV2-F8	TGAYACAGCTTAATCCRCTCAACAT	6775	25	60.8	40
hPIV2-R8	GGAGTTWCCGGCACARGTTATG	7846	22	61.2	54.5
hPIV2-F9	GRAGCGGGATCTATCAYCTAGGC	7710	23	61.4	52.2
hPIV2-R9	GCGGGAAGTGCCCTAGTAATAAG	8897	23	61.7	52.2
hPIV2-F10	AAGATTATRATATAGGCCAGAATGGC	8777	26	61.9	38.5
hPIV2-R10	CRATTAAATCTGGAGAAAGGTTGGA	9872	25	61.3	36
hPIV2-F11	TGGGTGTCTACAACTTAAAGATCCAG	9697	26	61.3	42.3
hPIV2-R11	ATGTATCATCAGGATCTGTGAGGTATG	10,772	27	60.8	40.7
hPIV2-F12	TCATTGCTTACTATGAGTCAAATTGG	10,610	26	60.2	34.6
hPIV2-R12	CGATATTGCGGTTAAATAATCTGC	11,675	24	60.7	37.5
hPIV2-F13	TCYATTCGTCAACTCACATATGATC	11,501	25	60.8	40
hPIV2-R13	CRATATCAAGTGCATCATTCCAGT	12,614	24	61	41.7
hPIV2-F14	AAGAAGAGTTGCATCAATGGCATA	12,484	24	61	37.5
hPIV2-R14	GCAAGAACTTGTTTAACYCCCCA	13,560	23	60.5	43.5
hPIV2-F15	AGACGTGCAATGAATCTTGATATTATC	13,445	27	60.7	33.3
hPIV2-R15	GTYGATTCGAGATCTATATGRACAAG	14,559	26	60.9	42.3
hPIV2-F16	WGCAGTTACRGACTTATCRACAAAGGA	14,461	27	62	37
hPIV2-R16	ACCAAGGGGAAAATCAATATGTTTT	15,654	25	61.9	32

* Nucleotide positions based on Washington/1964 prototype strain (GenBank accession number: NC_003461). ** Nucleotide positions based on Japan/1990 prototype strain (GenBank accession number: NC_003443).

## Data Availability

All sequences were submitted to Genbank (Accession Numbers: PP7577674-PP757767, PP886675-PP886722). Raw sequencing data are available in Sequence Read Archive (SRA) database BioProject ID: PRJNA1186829.

## Supplementary Materials

The supporting information can be downloaded at https://www.mdpi.com/article/10.3390/ijms252313119/s1.
