# Supplementary material for: Development of Primer Panels for Amplicon Sequencing of Human Parainfluenza Viruses Type 1 and 2
Source: Int J Mol Sci. 2024 Dec 6;25(23):13119. doi: 10.3390/ijms252313119 (PMC11641795; doi:10.3390/ijms252313119)
Supplement: Supplementary file 1 [file Supplementary_Figure_S1.pdf › Supplementary_Figure_S1.pdf]

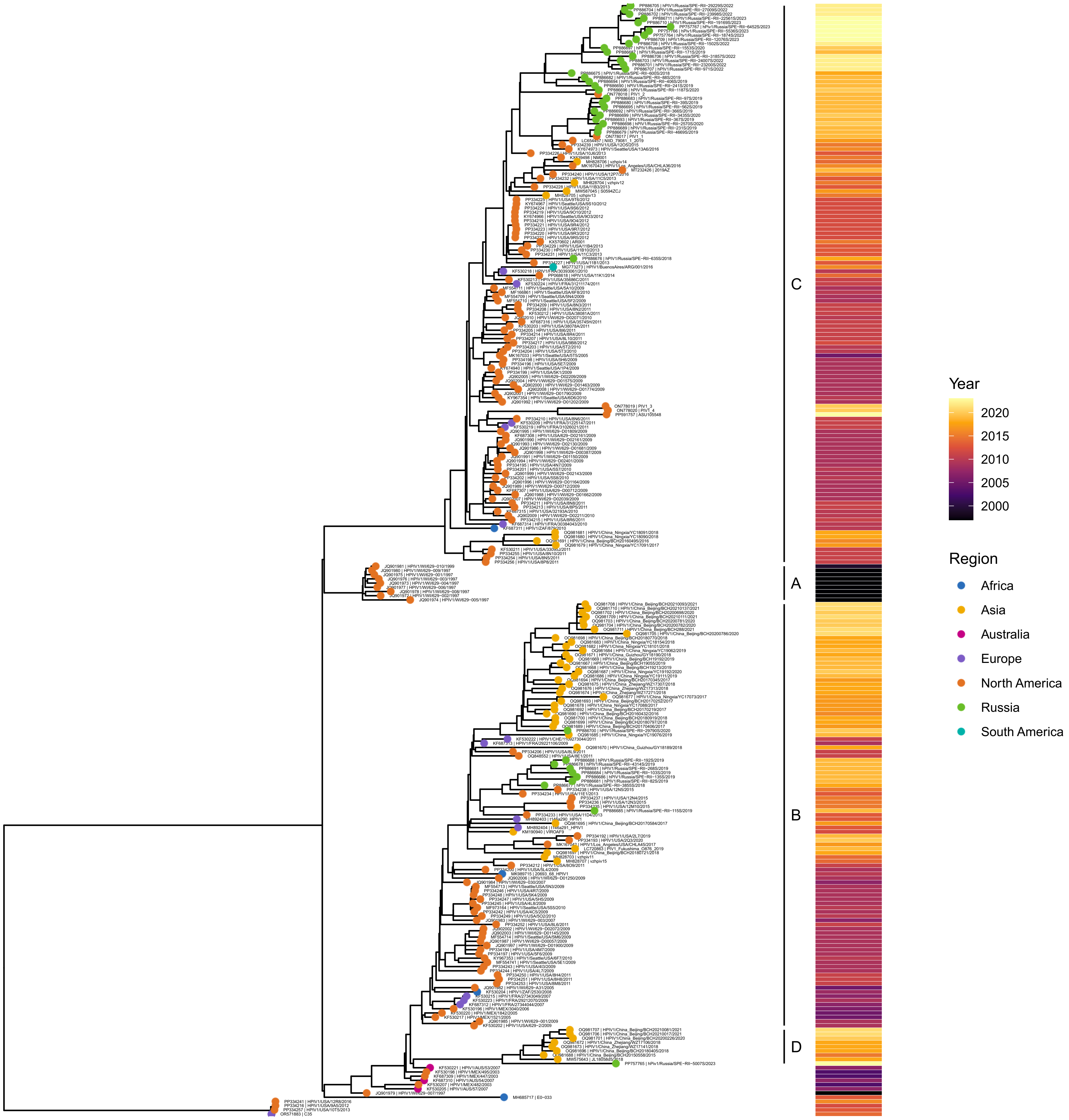

Supplementary Figure S1: Phylogenetic tree based on the whole-genome sequences of hPIV1. The phylogeny was reconstructed using the maximum likelihood (ML) method in the RAxML and visualized in R (ggtree). The four identified clades A, B, C and D are indicated. The names of strains and their corresponding accession numbers are provided. The Russian strains are labeled with green circles.
