# Supplementary material for: Development of Primer Panels for Amplicon Sequencing of Human Parainfluenza Viruses Type 1 and 2
Source: Int J Mol Sci. 2024 Dec 6;25(23):13119. doi: 10.3390/ijms252313119 (PMC11641795; doi:10.3390/ijms252313119)
Supplement: Supplementary file 1 [file Supplementary_Figure_S2.pdf › Supplementary_Figure_S2.pdf]

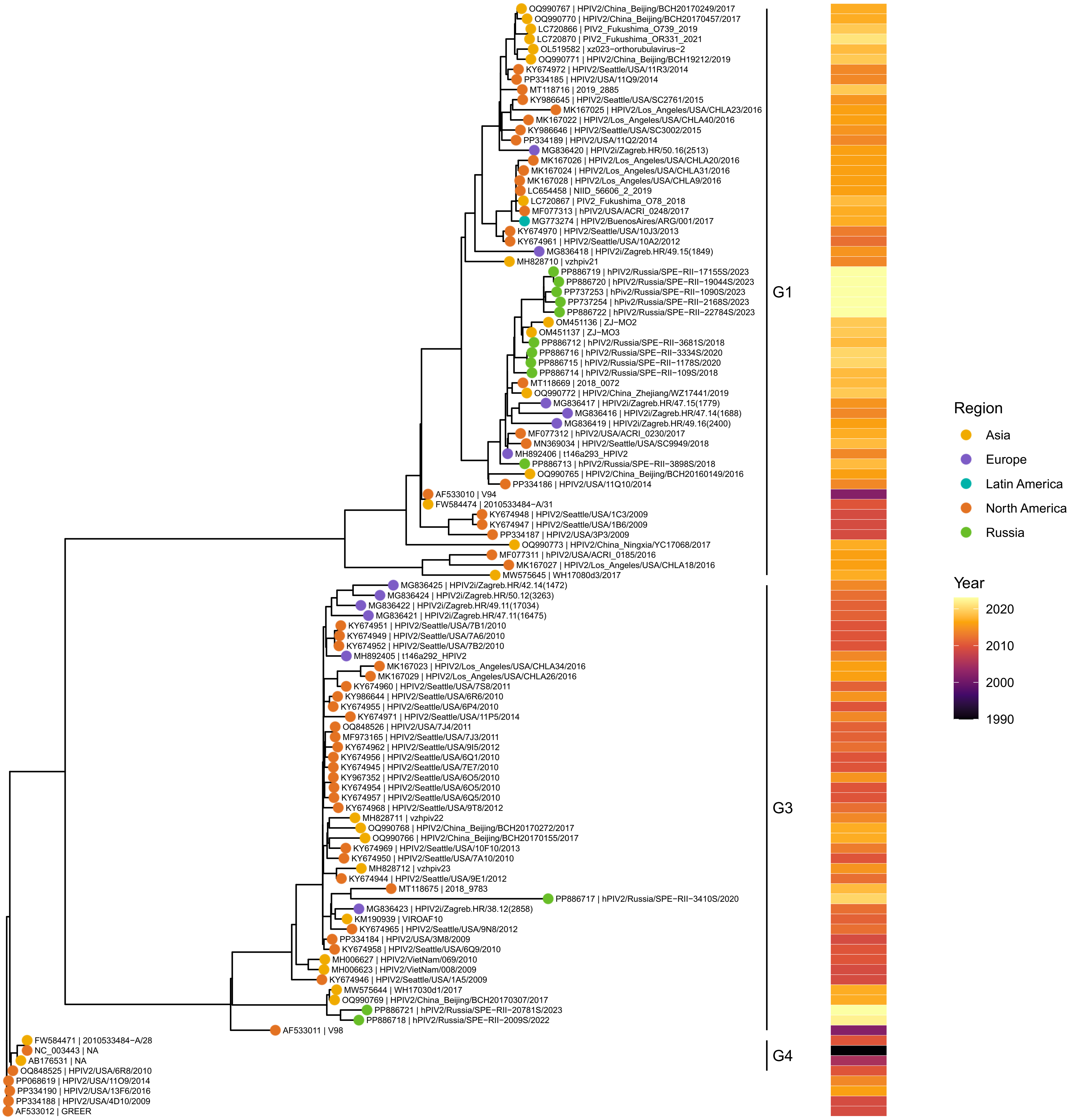

Supplementary Figure S2: Phylogenetic tree based on the whole-genome sequences of hPIV2. The phylogeny was reconstructed using the maximum likelihood (ML) method in the RAxML and visualized in R (ggtree). G1, G3 and G4 clades are indicated. The names of strains and their corresponding accession numbers are provided. The Russian strains are labeled with green circles.
